# Pit-1 inhibits BRCA1 and sensitizes human breast tumors to cisplatin and vitamin D treatment

**DOI:** 10.18632/oncotarget.3894

**Published:** 2015-05-08

**Authors:** Samuel Seoane, Efigenia Arias, Rita Sigueiro, Juan Sendon-Lago, Anxo Martinez-Ordoñez, Esteban Castelao, Noemí Eiró, Tomás Garcia-Caballero, Manuel Macia, Rafael Lopez-Lopez, Miguel Maestro, Francisco Vizoso, Antonio Mouriño, Roman Perez-Fernandez

**Affiliations:** ^1^ Department of Physiology, University of Santiago de Compostela, Santiago de Compostela 15782, Spain; ^2^ Center for Research in Molecular Medicine and Chronic Diseases-CIMUS, University of Santiago de Compostela, Santiago de Compostela 15782, Spain; ^3^ Department of Obstetrics and Gynecology, University of Santiago de Compostela, Santiago de Compostela 15782, Spain; ^4^ Department Organic Chemistry, Research Laboratory Ignacio Rivas, University of Santiago de Compostela, Santiago de Compostela 15782, Spain; ^5^ Oncology and Genetics Unit, Biomedical Research Institute of Vigo (IBIV), Complejo Hospitalario Universitario de Vigo, Servicio Galego de Saude (SERGAS), Vigo 36036, Spain; ^6^ Research Unit, Fundación Hospital de Jove, Gijón 33290, Spain; ^7^ Department of Morphological Sciences, University of Santiago de Compostela, Santiago de Compostela 15782, Spain; ^8^ Department of Clinical Oncology, University of Santiago de Compostela, Santiago de Compostela 15782, Spain

**Keywords:** breast cancer, Pit-1, BRCA1, cisplatin, vitamin D

## Abstract

The POU class 1 homeobox 1 (POU1F1, also known as Pit-1), pertaining to the Pit-Oct-Unc (POU) family of transcription factors, has been related to tumor growth and metastasis in breast. However, its role in response to breast cancer therapy is unknown.

We found that Pit-1 down-regulated DNA-damage and repair genes, and specifically inhibited BRCA1 gene expression, sensitizing breast cancer cells to DNA-damage agents. Administration of 1α, 25-dihydroxy-3-epi-vitamin D_3_ (3-Epi, an endogenous low calcemic vitamin D metabolite) reduced Pit-1 expression, and synergized with cisplatin, thus, decreasing cell proliferation and apoptosis *in vitro*, and reducing tumor growth *in vivo*. In addition, fifteen primary cultures of human breast tumors showed significantly decreased proliferation when treated with 3-Epi+cisplatin, compared to cisplatin alone. This response positively correlated with Pit-1 levels.

Our findings demonstrate that high levels of Pit-1 and reduced BRCA1 levels increase breast cancer cell susceptibility to 3-Epi+cisplatin therapy.

## INTRODUCTION

Breast cancer is currently treated according to the biological characteristics of the tumor (i.e. Luminal, HER2-enriched, basal-like, and normal breast-like) [1–2], which has permitted personalized therapies and improved clinical outcome. Research has led to additional classifications of breast cancer subtypes [3–6]. However, some tumors do not respond well to treatment, even those classified under subtypes with generally good prognosis. For example, some estrogen receptor (ER) positive tumors with high somatic mutation load are associated with poor overall survival [7]. HER2 tumor subtype also shows variable response to treatment depending on ER and progesterone receptor (PR) expression [8]. The basal-like subtype, often called triple-negative breast cancer (TNBC), usually has a bad prognosis because no targeted therapy exists for this subgroup of patients [4]. The basal-like subtype of tumors shows the highest frequency of DNA loss and gain compared with other subtypes [9]. In breast cancer, at least 35 significantly mutated genes, either by germline or somatic mutations, have been identified [10–11]. Half of these genes, including *BRCA1*, are associated with DNA-damage response [12]. BRCA1 is involved in the differentiation of breast epithelial cells [13], and its mutation impairs luminal progenitor cell differentiation and converts ER-positive luminal tumors into basal-like cancer [14]. Recently, it has been suggested that *BRCA1* gene heterozygosity suffices for cancer predisposition, by triggering genome instability that accumulates over many cell divisions [15].

Numerous studies have demonstrated that the 1α, 25-dihydroxyvitamin D_3_ (calcitriol, 1, 25D) hormone, which is the most biologically active form of vitamin D, regulates calcium and phosphorus homeostasis as well as other physiological and pathological processes including cell proliferation and differentiation. This has led to the study of its effects in cancer [16]. In human breast cancer cells, 1, 25D inhibits cycle progression, induces apoptosis, modulates cancer invasion and metastasis, and inhibits angiogenesis [17]. In addition, 1, 25D and its analogues can enhance, either synergistically or additively, the antitumor properties of several antineoplastic agents such as DNA-damaging agents (*i.e*., cisplatin) [18]. Nevertheless, its use in therapy has been limited because of its hypercalcemic side effects.

In breast cancer, previous studies have demonstrated that 1, 25D administration inhibits the Pit-1 transcription factor at the transcriptional level [19]. Pit-1 (also known as POU1F1) is expressed in the mammary gland [20], and its deregulation induces a malignant phenotype in breast cancer cells associated with tumor growth and metastasis [21]. However, its role in breast cancer response to therapy is unknown. The 1α, 25-dihydroxy-3-epi-vitamin D_3_ (3-Epi) is a low-calcemic vitamin D metabolite that was initially identified in the culture of human neonatal keratinocytes [22]. It has been shown that 3-Epi has similar biological activity to 1, 25D [23]. Therefore, this less calcemic vitamin D analogue, could be appropriate for treatment of breast tumor with elevated levels of Pit-1.

The present study evaluated the relationships between Pit-1 and BRCA1 in breast cancer, and the effect of the administration of cisplatin and 3-Epi in breast cancer cell lines, primary cultures of human breast tumors, and mice models. We show that Pit-1 inhibits BRCA1 gene expression and sensitizes breast cancer cells to DNA-damage agents, such as cisplatin. In addition, administration of 3-Epi synergizes with cisplatin, thus, decreasing cell proliferation and apoptosis *in vitro*, and reducing tumor growth *in vivo*. Our results reveal Pit-1 as a new marker for breast tumors susceptible to 3-Epi+cisplatin therapy.

## RESULTS

### Pit-1 sensitizes breast cancer cells to DNA-damage

**Figure 1:**
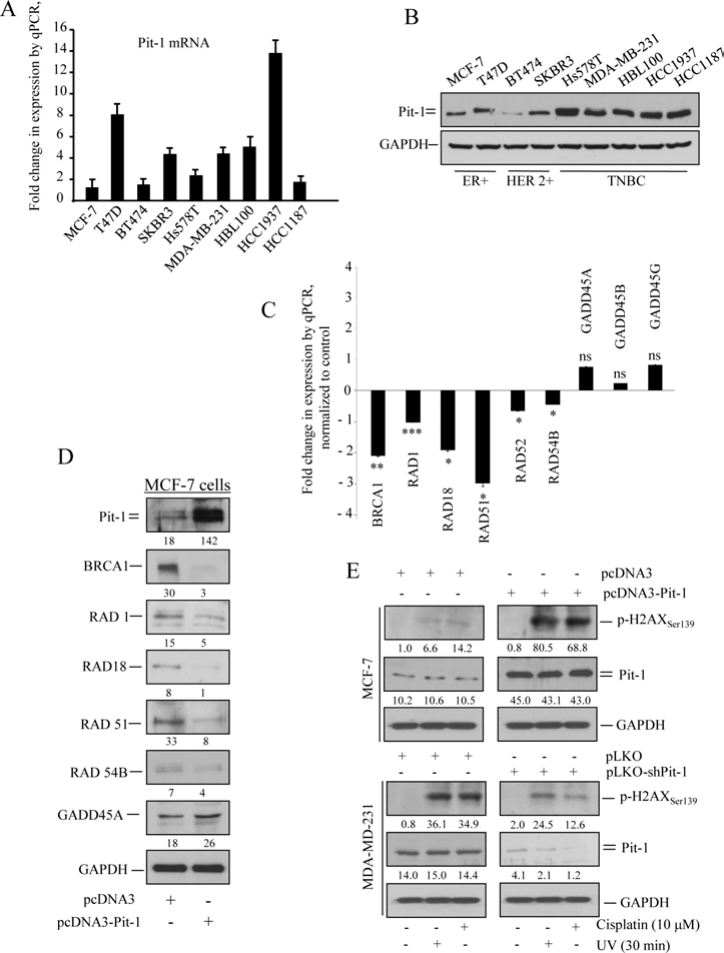
Pit-1 expression in breast tumor cell lines is linked to DNA damage response genes **A.** Real-time PCR of Pit-1 mRNA in the human breast tumor cell lines MCF7, T47D, BT474, SKBR3, Hs578T, MDA-MB-231, HBL100, HCC1937, and HCC1187. Pit-1 expression is plotted as mean ± SD of triplicates using *18S* gene expression as control. **B.** Western blot of Pit-1 protein from the breast tumor cell lines described above. **C.** Validation of microarray data in MCF-7-Pit-1 cells. mRNA levels were measured by qRT-PCR 48 h after transient Pit-1 overexpression. Fold-change with respect to levels in MCF-7 cells is represented. *** = *P* < 0.001, ** = *P* < 0.01, * = *P* < 0.05, ns = not significant. **D.** Representative Western blot of genes described in C. Values correspond to a densitometric analysis. **E.** Control (pcDNA3) and Pit-1 overexpressing (pcDNA3-Pit-1) MCF-7 cells, and control (pLKO-scrambled) and Pit-1 knocked-down (pLKO-shPit-1) MDA-MB-231 cells were treated with UV-light radiation or cisplatin to induce DNA-damage. Representative Western blot of phosphorilated-H2AX (p-H2AX_ser139_) as DNA-damage marker, Pit-1, and GAPDH proteins. Values correspond to a densitometric analysis.

Pit-1 expression levels in breast cancer cell lines were evaluated by real-time PCR and Western blot. Our data showed Pit-1 mRNA and protein expression in all breast cancer cell lines (Figure 1A–1B). Given that Pit-1 tends to have higher expression in aggressive cell lines, we carried out a microarray analysis, qRT-PCR, and Western blot in the low-aggressive MCF-7 (with low basal Pit-1 levels) cell line before and after Pit-1 overexpression. With respect to control MCF-7 cells, we found that Pit-1 overexpression significantly (*P* < 0.05) decreased DNA-damage response genes, such as *BRCA1*, and *RAD* family members, while it increased, but not significantly, DNA-damage sensor genes, such as *GADD45A* (Figure 1C–1D). Given that Pit-1 modify DNA-damage response/sensor genes, we further evaluated the role of Pit-1 on DNA-damage sensitivity. MCF-7 and MDA-MB-231 cells with low and high basal Pit-1 levels, respectively, were manipulated to induce Pit-1 overexpression or Pit-1 knockdown, and treated with the DNA-damage agent cisplatin (10 μM for 48 hours) or UV radiation, followed by Western blotting to determine phosphorylated histone H2AX (p-H2AX), a well known DNA double-strand break marker. Increased DNA-damage was found in cells with high Pit-1 levels after chemical and radiation challenge (Figure 1E).

### Pit-1 inhibits BRCA1 in breast cancer cells and human tumors

**Figure 2:**
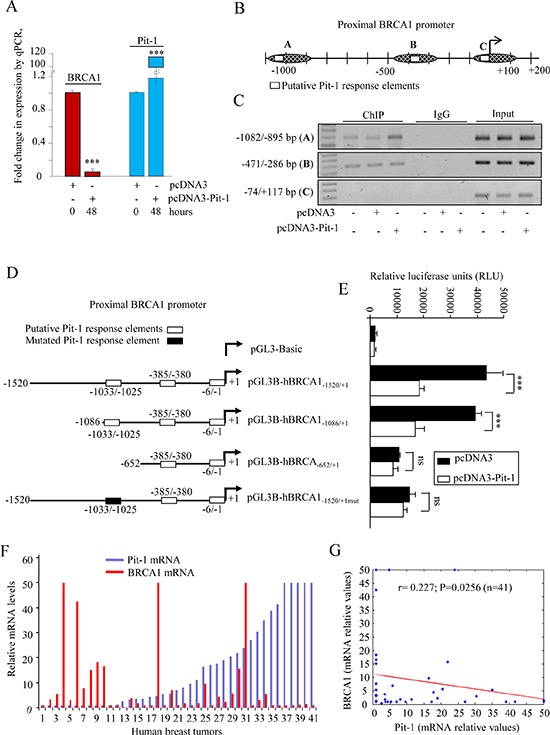
Pit-1 inhibits BRCA1 in breast cancer cells **A.** Quantitative PCR (qPCR) of BRCA1 and Pit-1 mRNA expression in control and Pit-1 overexpressing MCF-7 cells. BRCA1 and Pit-1 expression is plotted as mean ± SD of triplicates using *18S* gene expression as control. *** = *P* < 0.001. **B.** Diagram of the human *BRCA1* gene promoter showing the putative Pit-1 binding sites, and fragments amplified by primers used in the ChIP assay. **C.** Soluble chromatin prepared from control, pcDNA3, or pcDNA3-Pit-1 transfected MCF-7 cells was immunoprecipitated (IP) with an anti-Pit-1 antibody or control IgG. The IP DNA was amplified by PCR using primers (A, B, and C) that amplified regions of the BRCA1 promoter with the putative Pit-1 binding sites. **D–E.** BRCA1 promoter fragments were fused to the pGL3Basic vector (pGL3B) and transfected into MCF-7 cells. Then, cells were transfected with the pcDNA3 or the pcDNA3-Pit-1 overexpression vector. Normalized relative luciferase units (RLU) were calculated as the ratio of luciferase activity in the pcDNA3-Pit-1 transfected cells to that in the corresponding control (pcDNA3 transfected) cells. Data are plotted as mean ± SD of triplicates. *** = *P* < 0.001, ns = not significant. **F–G.** Pit-1 and BRCA1 mRNA was evaluated by qPCR in 41 breast tumor sample cDNA array. Dispersion plot indicates a significant negative correlation between Pit-1 and BRCA1 mRNA.

Given that Pit-1 reduced BRCA1 mRNA and protein expression (Figure 1C–1D), and that BRCA1 is a key protein in DNA-damage response, we evaluated the role of Pit-1 in BRCA1 regulation. Real-time PCR showed significantly (*P* < 0.001) decreased BRCA1 mRNA levels after Pit-1 overexpression in MCF-7 cells (Figure 2A). Pit-1 regulated BRCA1 at transcriptional level in MCF-7 cells, as shown by chromatin immunoprecipitation (ChIP) (Figure 2B–2C) and luciferase reporter assays (Figure 2D–2E). We found specific Pit-1 binding to the position located between −1025 to −1033 base pairs (bp) from the start transcription site in the BRCA1 gene promoter, as demonstrated by site-directed mutagenesis (Figure 2D–2E).

Pit-1, BRCA1 and 18S mRNA expression were evaluated by real-time PCR on a cDNA microarray to explore the relationship between Pit-1 and BRCA1 in human breast tumors (*n* = 41) (Figure 2F). A significantly (*r* = 0.227, *P* = 0.025) negative correlation between Pit-1 and BRCA1 mRNA expression was found (Figure 2G).

### 3-Epi inhibits Pit-1 expression in breast

**Figure 3:**
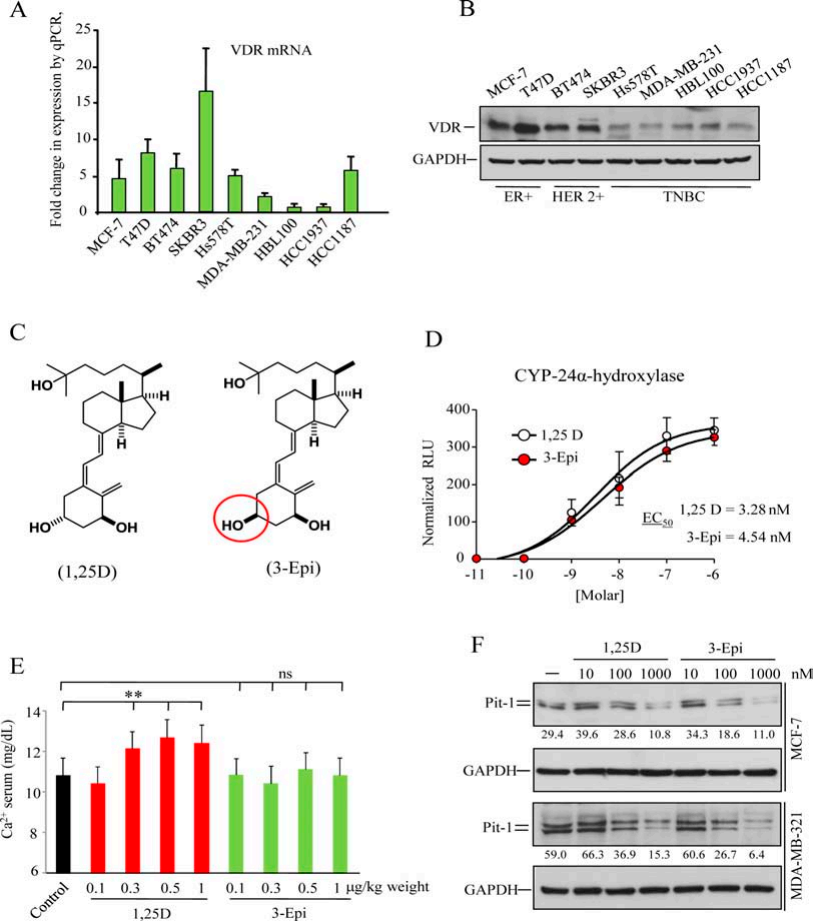
Vitamin D receptor (VDR) expression in human breast cell lines, and biological activity of the vitamin D derivative 1α, 25-dihydroxy-3-epi-vitamin D3 (3-Epi) **A.** Real-time PCR of VDR mRNA in the human breast tumor cell lines MCF7, T47D, BT474, SKBR3, Hs578T, MDA-MB-231, HBL100, HCC1937, and HCC1187. VDR expression are plotted as mean ± SD of triplicates using *18S* gene expression as control. **B.** Western blot of VDR protein from the breast tumor cell lines described above. **C.** Structure of 1, 25D and 3-Epi. **D.** Transcriptional activation of the 24-hydroxylase gene (*CYP24A1*) by 1, 25D and 3-Epi. MCF-7 cells were transfected with the pCYP24A1-luc vector (encoding the luciferase gene under control of a consensus vitamin D response element) and treated with 1, 25D or 3-Epi (10^−11^ to 10^−6^ M) for 24 h. The EC_50_ values derived from dose-response curves, and represent the analogue concentration capable of increasing luciferase activity by 50%. Error bars represent standard deviation (SD). **E.** Serum calcium level in mice treated with 1, 25D and 3-Epi. Five mice per group were treated with 0.1, 0.3, 0.5, and 1 μg/kg weight of 3-Epi, 1, 25D, or vehicle (sesame oil) every other day for 3weeks, and calcium levels were measured on day 21. Error bars represent standard deviation (SD). ** = *P* < 0.01, ns = not significant. **F.** MCF-7 and MDA-MB-231 cells were treated with 1, 25D and 3-Epi (10^−8^ to 10^−6^ M) for 48 h. Representative immunoblot of Pit-1 and GAPDH. Values correspond to a densitometric analysis.

Vitamin D has been related to anti-tumoral effects, and this hormone mediates by binding to the vitamin D receptor (VDR). Therefore, VDR expression levels in breast cancer cell lines were evaluated by real-time PCR and Western blot. We found VDR expression in all cell lines evaluated, as previously demonstrated [24] (Figure 3A–3B). Given that the use of 1, 25D in therapy is limited because of its hypercalcemic side effects, we tested to see if the 3-Epi vitamin D derivative (Figure 3C) had similar biological properties. We carried out a luciferase gene reporter assay and *in vivo* calcemic analysis in mice. Both 3-Epi and 1, 25D regulated the *CYP24A1* gene, a classic 1, 25D target with similar EC_50_ (Figure 3D). However, no significant hypercalcemic activity was observed in mice treated with 3-Epi at doses of 1 μg/kg weight (Figure 3E). MCF-7 and MDA-MB-231 cells were also treated with 1, 25D or 3-Epi (10 to 1000 nM), and Pit-1 was evaluated by Western blot. Importantly, both 3-Epi and 1, 25D at 100 and 1000 nM reduced basal Pit-1 expression (Figure 3F), as previously demonstrated for 1, 25D [19].

### 3-Epi synergizes with cisplatin in Pit-1 sensitized cells

**Figure 4:**
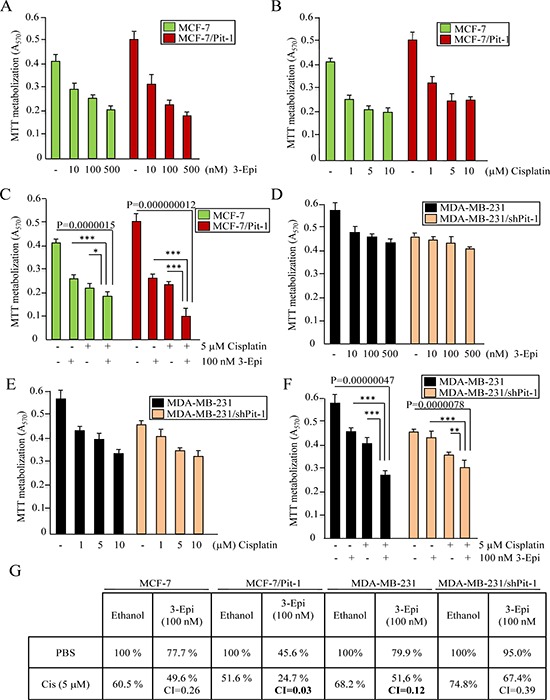
Pit-1 modifies cell proliferation response to 3-Epi and cisplatin in breast cancer cells **A–F.** Control and Pit-1 overexpressing MCF-7 cells (MCF-7/Pit-1), and control and Pit-1 knocked down MDA-MB-231cells (MDA-MB-231/shPit-1) were cultured in presence of ethanol, 3-Epi (10, 100 and 500 nM), cisplatin (1, 5 and 10 μM), and 100 nM of 3-Epi + 5 μM of cisplatin. Two days later, cell proliferation was evaluated by MTT assay. Values are plotted as mean ± SD from two experiments performed in quadruplicate. * = *P* < 0.05, ** = *P* < 0.01, *** = *P* < 0.001. **G.** Cell proliferation percentages after treatment with 100 nM of 3-Epi, 5 μM of cisplatin, and 100 nM of 3-Epi + 5 μM of cisplatin in breast cancer cells as described above. Percentage values of ethanol-treated samples were taken as 100%. CI values < 1.0 indicate synergism.

Using breast cancer cell lines with different levels of Pit-1 (MCF-7, MCF-7/Pit-1, MDA-MB-231, and MDA-MB-231/shPit-1), which therefore had different sensitivity to DNA-damage agents (see Figure 1E), MTT assays were performed to evaluate cell proliferation after treatment with 3-Epi, cisplatin, and both together. Proliferation response to 3-Epi and cisplatin was better (reduced proliferation) in cells with high Pit-1 expression (Figure 4A–4F). Our data also indicated synergy in cells treated with 3-Epi at doses of 100 nM and 5 μM cisplatin, and this synergy was higher in cells with elevated Pit-1 expression (combination index (CI): 0.03 in MCF-7/Pit-1 cells, and 0.12 in MDA-MB-231 cells) (Figure 4C, and 4G). Proliferation percentages in control and treated cells, as well as the CI are shown in Figure 4G.

**Figure 5:**
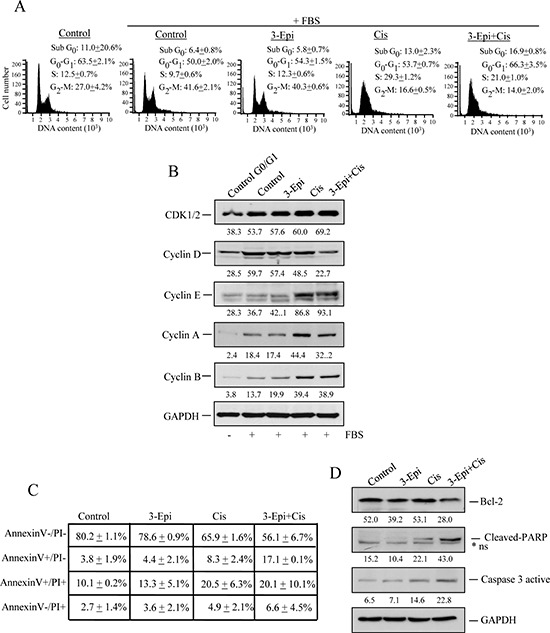
3-Epi enhances cisplatin effect in Pit-1-overexpressed breast cancer cells **A.** MCF-7/Pit-1 cells were serum-starved for 24 hours and treated with the indicated drugs (100 nM of 3-Epi, 5 μM of cisplatin, or a combination of both drugs at same dose) in complete medium for 24 hours, and cell cycle was examined by flow cytometry after staining with propidium iodide (PI). A representation of three independent experiments is shown. **B.** MCF-7/Pit-1 cells were treated with the indicated drugs (as above) for 24 hours, and an immunoblot analysis was done for cell cycle (CDK1/2, cyclin D, cyclin E, cyclin A, and cyclin B) proteins. GAPDH was used as loading control. **C.** Flow cytometry analysis of apoptosis in MCF-7/Pit-1 cells treated with the indicated drugs for 48 hours and stained with Annexin V and PI. Table shows the percentage of cells stained with Annexin V and/or PI plotted as mean ± SD. **D.** MCF-7/Pit-1 cells were treated with the indicated drugs for 48 hours as described above, and an immunoblot analysis was done for anti-apoptotic (Bcl-2) or pro-apoptotic (cleaved-PARP and caspase 3 active) proteins. GAPDH was used as loading control. *ns = not specific.

To evaluate why combination of 3-Epi+cisplatin synergistically reduced cell proliferation in cells with Pit-1 overexpression, we first evaluated cell cycle. MCF-7/Pit-1 cells were treated with 3-Epi, cisplatin, and 3-Epi+cisplatin, and subjected to flow cytometry using propidium iodide (PI). 3-Epi did not significantly modify cell cycle, in relation to control cells. As expected, cisplatin reduced G_2_-M and increased S phases of cell cycle with respect to control and 3-Epi-treated cells. The combination of both drugs increased the G_0_–G_1_ phase as compared to cisplatin administration alone (Figure 5A). Western blot showed that 3-Epi+cisplatin reduced cyclin D expression as compared to cisplatin alone, which seems to delay entry of cells into G1 and S phases, as shown by reduced expression of cyclin A (Figure 5B). To study the effect of 3-Epi and cisplatin on apoptosis, MCF-7/Pit-1 cells were treated, stained with AnnexinV-FITC and PI, and analyzed by flow cytometry. A significantly (*P* < 0.001) increased rate of early apoptosis (annexin V+/PI-) was observed in 3-Epi+cisplatin-treated cells, compared to cells treated with cisplatin alone (Figure 5C, and Supplementary Figure S1B). Protein analyses demonstrated increased levels of cleaved PARP and active caspase 3, and decreased levels of the anti-apoptotic Bcl-2 protein after 3-Epi+cisplatin treatment, compared to cisplatin alone (Figure 5D, and Supplementary Figure S1C).

**Figure 6:**
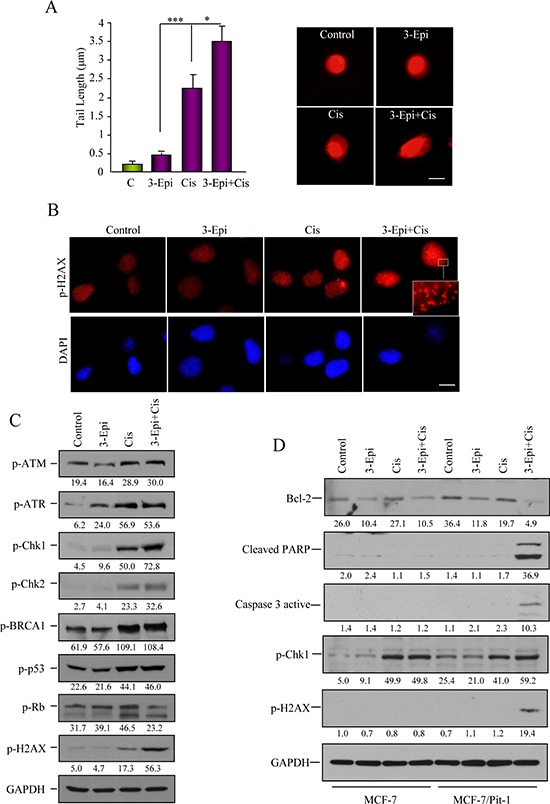
3-Epi enhances cisplatin effect in Pit-1-sensitized breast cancer cells **A.** Pit-1 overexpressing MCF-7 cells (MCF-7/Pit-1) treated with ethanol, 100 nM of 3-Epi, 5 μM of cisplatin, and 100 nM of 3-Epi + 5 μM of cisplatin were analyzed for DNA-damage in a comet assay. DNA-damage induced by each treatment is indicated by average tail length. Data are represented as mean ± SD. A representative image is shown in B. Scale bar: 10 μm. **B.** Immunofluorescence of p-H2AX protein, as marker of DNA-damage, after treatment with ethanol, 3-Epi, cisplatin, and 3-Epi+cisplatin in MCF-7/Pit-1 cells. Scale bar: 10 μm. Cisplatin and 3-Epi+cisplatin modify DNA-damage phosphorylated proteins. **C.** Immunoblot analysis of extracts in the conditions described above was done for the DNA-damage phosphorylated (p) proteins, p-ATM_Ser1981_, p-ATR_Ser428_, p-Chk1_Ser296_, p-Chk2_Thr68_, p-BRCA1_Ser988_, p-p53_Ser15_, p-Rb_Ser139_, and p-H2AX_Ser139_. GAPDH was used as loading control. Values correspond to a densitometric analysis. **D.** Immunoblot analysis of Bcl-2, cleaved PARP, caspase 3 active, p-Chk1_Ser296_, p-H2AX_Ser139_, and GAPDH (used as loading control) in MCF-7 and MCF-7/Pit-1 cells treated with ethanol, 100 nM of 3-Epi, 5 μM of cisplatin, and 100 nM of 3-Epi + 5 μM of cisplatin. Values correspond to a densitometric analysis.

To assess if 3-Epi+cisplatin produced more DNA damage than cisplatin alone, DNA fragmentation and DNA damage-induced proteins were evaluated. By comet assay we showed that 3-Epi+cisplatin treatment in MCF-7/Pit-1 cells induced a significantly (*P* < 0.05) longer trailing tail, indicating increased DNA fragmentation (Figure 6A). Immunofluorescence analysis of p-H2AX showed increased DNA damage after 3-Epi+cisplatin as compared to cisplatin alone (Figure 6B). Proteins involved in DNA damage and repair were also evaluated by Western blot in MCF-7/Pit-1 cells. Figure 6C shows high basal phosphorylation levels for ATM, BRCA1, and p53, as well as increased ATM, ATR, Chk1, Chk2, BRCA1, p53, Rb, and H2AX phosphorylation in cisplatin-treated cells. Treatment with 3-Epi+cisplatin increased p-Chk1 and p-H2AX proteins, with respect to cisplatin alone. To further compare the effect of treatments on MCF-7/Pit-1 (overexpressing Pit-1) and wild MCF-7 cells, a Western blot of Bcl-2, cleaved PARP, caspase 3 active, p-Chk1, and p-H2AX proteins was also carried out. Figure 6D shows that high levels of Pit-1 potentiate the effect of 3-Epi+cisplatin treatment.

### Cisplatin+3-Epi reduced breast tumor growth

**Figure 7:**
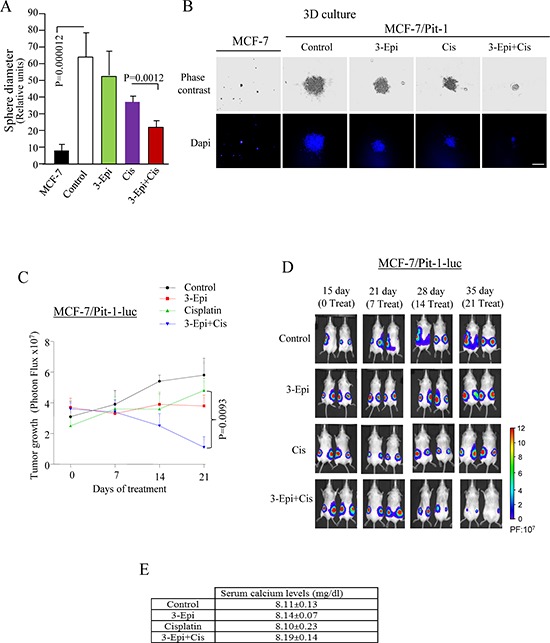
3-Epi potentiates cisplatin to reduced tumor growth **A.** Three-dimensional cultures of MCF-7 and MCF-7/Pit-1 cells were treated for 7 days with ethanol, 100 nM of 3-Epi, 5 μM of cisplatin, and 100 nM of 3-Epi + 5 μM of cisplatin, stained with DAPI, followed by sphere diameter quantification. Twenty spheres were scored for each condition. MCF-7 cells (without Pit-1 overexpression) do not show 3D growth after 7 days of culture. **B.** Representative example of A. Scale bar = 150 μm. **C.** 24 SCID mice were subcutaneously injected with MCF-7/Pit-1-luc cells (left and right flanks). Fifteen days later (day 0 of treatment), mice were split up into 4 groups and injected intraperitoneally (i.p.) with one of the following: a) 3-Epi every other day (0.5 μg/kg weight, dissolved in sesame oil), b) twice with cisplatin (7 mg/kg weight, i.p., days 0 and 7 of treatment), c) 3-Epi every other day (i.p., 0.5 μg/kg weight) + cisplatin twice (7 mg/kg weight, i.p., days 0 and 7 of treatment), or d) sesame oil (control group). On day 36 (day 21 of treatment), mice were sacrificed. Tumor growth was monitored every 7 days from day 15 until day 36 using *in Vivo* Imaging System. **D.** Representative image of mice described in C. Panel in D indicates bioluminescence intensity (PF = Photon flux). **E.** Treatment with 3-Epi or 3-Epi+cisplatin during 21 days as in (C) does not increase serum calcium levels.

To explore the effect of 3-Epi and cisplatin on tumor growth *in vitro*, MCF-7/Pit-1 cells were cultured in matrigel. After forming three-dimensional (3D) cultures, cells were treated for 4 days. Administration of cisplatin+3-Epi significantly (*P* < 0.01) enhanced the effect of cisplatin, thus, decreasing culture growth (Figure 7A–7B). To further evaluate the effect of both drugs on tumor growth *in vivo*, we used the SCID mouse tumor xenograft model. SCID mice were subcutaneously injected on day 0 with MCF-7/Pit-1 cells, and fifteen days later treated intraperitoneally (i.p) with sesame oil, 3-Epi, cisplatin, and 3-Epi+cisplatin. Tumor growth at day 35 (21 days after treatment) was significantly lower in mice injected with MCF-7/Pit-1 cells and treated with 3-Epi+cisplatin as compared to cisplatin alone (*P* = 0.0093) (Figure 7C–7D). Representative images of treated mice are shown in Figure 7D. Calcium in serum was also determined at day 35 in MCF-7/Pit-1 injected mice. Mice treated with 3-Epi and 3-Epi+cisplatin showed no hypercalcemia (Figure 7E).

### 3-Epi improves cisplatin treatment in human breast tumors with high Pit-1 levels

**Figure 8:**
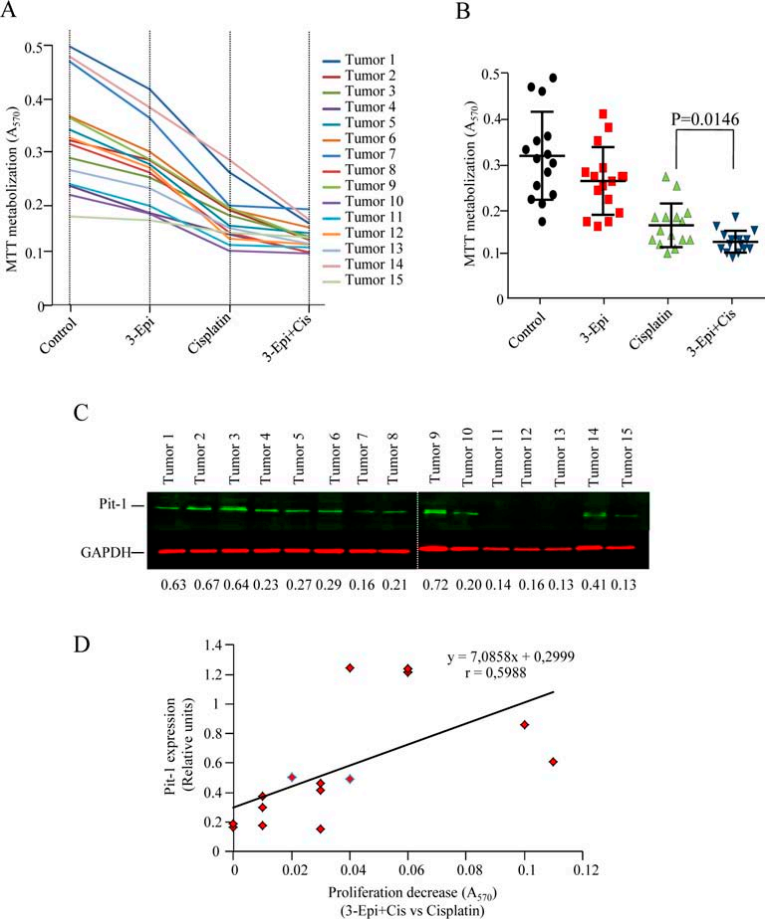
Cell proliferation response to 3-Epi+cisplatin treatment in primary cultures of breast tumors is related to Pit-1 levels **A.** Fifteen primary cultures of human breast tumors (BT) were treated for 48 h with ethanol (controls), 100 nM of 3-Epi, 5 μM of cisplatin, or 100 nM of 3-Epi + 5 μM of cisplatin. Then, MTT was added and absorbance measured at 570 nm. Absorbance values are plotted as the mean of quadruplicate values. **B.** Cell proliferation response in primary breast cultures was significantly (*P* = 0.014) reduced after 3-Epi+cis as compared with cisplatin. **C.** Representative Western blot of Pit-1 levels in primary breast tumors. Pit-1 expression was evaluated by quantitative Western blot. Values were corrected by GAPDH expression. **D.** Statistical analyses indicated that reduced proliferation response to 3-Epi+cis as compared to cisplatin alone is correlated with Pit-1 levels.

Given that proliferation response to 3-Epi and cisplatin seems to be related to Pit-1 levels in breast cancer cell lines, fifteen primary cultures from human breast tumors were used to evaluate the effect of 3-Epi and cisplatin (Supplementary Table S1). Cultures were untreated (Control), treated with 3-Epi (100 nM), cisplatin (5 μM), or 3-Epi+cisplatin (100 nM+5 μM, respectively), and an MTT assay was then performed to evaluate cell proliferation. Pit-1 protein expression was also evaluated in each tumor by quantitative Western blot. All tumors showed reduced proliferation rates after treatment with 3-Epi, cisplatin and 3-Epi+cisplatin (Figure 8A and Supplementary Figure S2A–S2B). In addition, a significant (*P* = 0.014) decrease in proliferation was observed after treatment with 3-Epi+cisplatin vs. cisplatin alone (Figure 8B). Pit-1 protein expression was also quantified in primary tumors by Western blot using an Odissey Imager (Figure 8C). Statistical analyses showed a positive correlation (*r* = 0.5988) between Pit-1 expression and the response to 3-Epi+cisplatin versus cisplatin alone (Figure 8D).

## DISCUSSION

Here we show that Pit-1 down-regulates DNA-damage and repair genes. In particular, Pit-1 negatively regulates the *BRCA1* gene at transcriptional level, and sensitizes breast cancer cells to DNA-damage agents. Administration of the vitamin D derivative 3-Epi to breast cancer cells synergizes with cisplatin, thus, increasing accumulation of cells at G0–G1 phase of cell cycle, DNA damage and apoptosis, and decreasing tumor growth. In addition, primary cultures of human breast tumors treated with 3-Epi+cisplatin presented significantly less proliferation as compared to treatment with each drug alone, and response was positively related to Pit-1 expression levels.

Our first objective was to evaluate Pit-1 expression in human breast cancer cell lines. Pit-1 mRNA and protein was present in all cells studied, with higher Pit-1 expression in the most aggressive cells. To determine Pit-1-induced genomic effects in breast cancer cells, microarray analyses and Western blots were carried out on the low-aggressive MCF-7 cell line before and after Pit-1 overexpression. Pit-1 reduced BRCA1 and RAD family members of DNA-repair proteins. All of this seems to sensitize breast cancer cells to DNA-damage agents. In fact, higher levels of Pit-1 were related to higher sensitization to DNA-damage agents, such as ultraviolet light radiation or cisplatin treatment.

Given that BRCA1 protein has a tumor-suppressive role and is considered to be a “chromosome custodian” [15], we delved into the relationships between Pit-1 and BRCA1. We found that Pit-1 negatively regulated BRCA1 transcription in breast cancer cells, and that Pit-1 inversely correlated with BRCA1 mRNA levels in human breast tumors samples, which is in line with other microarray datasets [25–27]. Reduction of BRCA1 levels in mammary epithelial cells has been found to enhance cell proliferation and down-regulate differentiation genes [13]. It has also been found that treatment with a retroviral vector expressing wild-type BRCA1 significantly inhibited tumor growth and increased survival in mice with established MCF-7 tumors [28]. Given that Pit-1 down-regulates DNA-damage and repair genes (i.e. BRCA1) and sensitizes breast cancer cells to DNA-damage agents, in this study we used the antineoplastic drug, cisplatin, which is frequently applied to BRCA1-associated breast tumors [29].

Human breast tumors have recently been classified by Santagata et al. [6] according to vitamin D, androgen, and estrogen hormone receptor expression, suggesting that combining VDR agonists (i.e. 1, 25D or analogues) with standard chemotherapy treatment could inhibit proliferation more effectively than chemotherapy alone [6]. Therefore, we combined cisplatin with 3-Epi, a 1, 25D natural derivative [30]. 1, 25D or some of its analogues have been combined with cisplatin and other chemotherapeutic agents for the treatment of breast cancer, showing synergism and potentiation of cisplatin effect [17–18, 31–32]. However, the anti-tumor results of this approach have been disappointing probably because hypercalcemia concerns have led to the use of 1, 25D in low doses [33–34]. Our data indicates that 3-Epi has similar biological properties without inducing hypercalcemia at doses at least three times higher than 1, 25D. In addition, 3-Epi has a greater metabolic stability than 1, 25D because its inactivation by the CYP24A1 catabolic enzyme is much slower [35]. Here, we demonstrate that 3-Epi reduces Pit-1 expression in MCF-7 cells, as demonstrated previously with 1, 25D [19]. Treatment of MCF-7/Pit-1 and MDA-MB-231 cell lines with 3-Epi+cisplatin showed synergy with respect to cisplatin alone. Combined treatment decreased cell proliferation and cyclin D expression, arresting cells at G_0_ phase of cell cycle, as shown by reduced cyclin A expression, and increased apoptosis.

In addition, comet assay, and p-H2AX immunofluorescence analyses demonstrated that 3-Epi+cisplatin significantly increased DNA damage with respect to either drug alone. Our results clearly demonstrated that the 3-Epi+cisplatin combination was more effective in reducing tumor growth than administration of either drug alone. Finally, using primary cultures of human breast tumors, and given that Pit-1 protein levels seem to be a better marker for 1, 25D-sensitivity than Pit-1 mRNA levels, we evaluated cell proliferation after 3-Epi, cisplatin, and 3-Epi+cisplatin treatment, and correlated the effect on cell proliferation with Pit-1 protein expression. Cell proliferation was significantly decreased after 3-Epi+cisplatin treatment as compared to cisplatin alone, and this effect was positively related to Pit-1 expression.

Our data correlate with previous studies showing better response to cisplatin treatment in breast cancer patients with low BRCA1 levels [29, 36], and studies showing increased BRCA1 expression in breast cancer cells after treatment with 1, 25D or vitamin D analogues [37–38]. Recently, BRCA1 and VDR association has been demonstrated to activated genes involved in anti-tumor effects of vitamin D, and that a complete knockdown of BRCA1 abolishes the effects of vitamin D analogues [39]. Our data also demonstrate that Pit-1 levels are important to predicting treatment response, and, that administration of 3-Epi+cisplatin in patients with elevated Pit-1 levels could improve clinical outcomes in relation to cisplatin alone. In addition, a recent study by Huang et al. [40] has demonstrated that Pit-1 binds and represses MRE11 gene expression, sensitizing breast cancer cells to chemotherapeutic treatments. Although we cannot conclude from our data that MRE11 is involved in the sensitization of breast cancer cells to 3-Epi+cisplatin in Pit-1 overexpressed cells, our findings are in line with those observed by Huang et al. Further studies are necessary to confirm this data.

In summary, our study demonstrates that Pit-1 transcriptionally represses BRCA1 expression, and sensitizes breast cancer cells to combined treatment with cisplatin and the low calcemic 3-Epi vitamin D metabolite.

## MATERIALS AND METHODS

### Reagents

1, 25D and 3-Epi were synthesized at the Department of Organic Chemistry (University of Santiago de Compostela, Spain) as detailed in Supporting Information. Cisplatin was obtained from Ferrer Farma laboratories (Barcelona, Spain).

### Cell lines, primary cultures, treatments, and breast tumors cDNA samples

The human breast adenocarcinoma cell lines MCF-7, T47D, Hs578T and MDA-MB-231 were obtained from European Collection of Cell Culture (ECACC; Porton Down, UK). The human breast adenocarcinoma cell lines BT474, SKBR3, HCC1937 and HCC1187 were obtained from American Type Culture Collection (ATCC; Manassas, USA). The HBL100 breast cancer cell line was obtained from Cell Lines Service (CLS; Eppelheim, Germany). All cells were grown as previously described [21]. Three-dimensional cultures, isolation and culture of primary breast tumors are described in supplementary methods. Patient and tumor characteristics are shown in Supplementary Table S1. Breast cancer cell lines and primary cultures were treated for 48 h with ethanol (control cells), 3-Epi (10, 100 and 500 nM), cisplatin (1, 5, and 10 μM), and 3-Epi+cisplatin (100 nM+5 μM) in the MTT assay. In all other *in vitro* experiments, 3-Epi was used at 100 nM, cisplatin at 5 μM, and 3-Epi+cisplatin at doses of 100 nM+5 μM, respectively, unless specifically indicated. Cisplatin and 3-Epi were diluted in PBS and ethanol, respectively. Treatment of cells with UV radiation was carried out by exposure to 150 J/m2 of UV radiation for 30 min. Pit-1 and BRCA1 mRNA expression was evaluated by real-time PCR in human breast tumors (*n* = 41, Tissue Scan cDNA array, Origene, Rockville, USA).

### RNA isolation, real-time PCR, mRNA microarray, and Western blot

Total RNA from the cell lines and primary cultures was isolated with TRIzol reagent (Invitrogen, Barcelona, Spain). cDNA synthesis was performed as described elsewhere [21]. Pit-1, BRCA1, RAD1, RAD18, RAD51, RAD52, RAD54B, GADD45A, GADD45B, GADD45G, vitamin D receptor (VDR) and 18S mRNA levels were quantified using real-time PCR. Microarray assay of mRNA was performed using an Affymetrix Human Gene 1.0 ST Array (GEO database access no. GSE64101). Western blotting was performed as previously described [21]. Relative protein expression was quantified using ImageJ software (National Institutes of Health, Bethesda, USA). Pit-1 quantification in primary cultures of human breast tumors was performed using the LI-COR Odyssey software (Homburg, Germany). Primers and antibodies are described in Supporting Information.

### Plasmids, transfections, and luciferase reporter and chromatin immunoprecipitation (ChIP) assays

Transient transfections were performed in MCF-7 cells using the pcDNA3-Pit-1 overexpression vector and the pcDNA3 empty vector as control. Stable transfection of Pit-1 into MCF-7 cells (MCF-7/Pit-1) was performed as previously described [21] and briefly explained in Supplemental Information. Stable clones of Pit-1 knock-down in MDA-MB-231 cells (MDA-MB-231/shPit-1) were performed as described in Supplemental Information. Stable Pit-1 overexpressing MCF-7 (MCF-7/Pit-1) cells used for *in vivo* experiments were transfected with pBABE-puro-Luc vector and selected with puromicin to obtain MCF-7/Pit-1-luc cells. For luciferase reporter assay, MCF-7 cells were cultured as described above, and 12–24 h before transfection 2 × 10^5^ cells per well were seeded in 24-well plates and allowed to attach overnight. Cells were then transfected with the pCYP24A1-luc vector, pcDNA3, pcDNA3-Pit-1 and pGL3B-hBRCA1 constructs (pGL3B-hBRCA1_−1520/+1_, pGL3B-hBRCA1_−1086/+1_, pGL3B-hBRCA1_−652/+1_, and the mutant pGL3B-hBRCA1_1520/+1mut_vector) using JetPEI transfection reagent (PolyPlus Transfection, Illrich, France). The proximal promoter regions of the human BRCA1 gene constructs, and site-directed mutagenesis was carried out as described in Supplemental Information. The pCYP24A1-luc vector (1 μg, kindly provided by Dr. Aranda, Madrid) encodes the luciferase gene under control of a consensus vitamin D response element (CYP24A1), and is very responsive to 1, 25D treatment.

ChIP assay was carried out in control and Pit-1 overexpressing MCF-7cells. Briefly, cells were fixed for 10 min with paraformaldehyde, washed with PBS, lysed andsonicated. Diluted soluble chromatin fractions were immunoprecipitated with 1 μg polyclonal anti-Pit-1 antibody (Santa Cruz Biotechnologies) or control human IgG (Sigma Aldrich, Madrid, Spain). The histone-DNA crosslinks were reversed by 4 h incubation at 65°C. The DNA from these samples was extracted through phenol/chloroform and ethanol precipitated with 20 μg of glycogen. The DNA extracted was then dissolved in 30 μl of H_2_O. PCR was used to analyze the DNA fragment from the ChIP assay. Five microliters of assayed DNA (ChIP sample) and 5 μl of input/start material were used in each 50-μl reaction. Primers and PCR procedures are detailed in Supporting Information.

### Cell proliferation, cell cycle, apoptosis, immunofluorescence (IF) and comet assays

Cell proliferation assays were carried out by MTT assay, as previously described [41].

In order to determine whether the combination of 3-Epi and cisplatin was additive, antagonist, or synergistic, we used the CalcuSyn v2.0 software programme (Biosoft, Ferguson, USA) as previously described [42]. This program allows the calculation of the combination index (CI) based on the algorithm of Chou and Talalay. Combination index values less than 1 indicate synergism, values equal to 1 indicate an additive effect, whereas values greater than 1 indicate antagonism. Combination index values from three independent experiments were generated. Results were plotted as the mean values of duplicates from two independent experiments.

For cell cycle analyses MCF-7/Pit-1 cells were cultured, treated with different drugs for 24 hours, and stained with propidium iodide (PI). For apoptosis analyses, MCF-7/Pit-1 cells were incubated in the dark with Annexin V-FITC (BD Biosciences, San Jose, USA), and PI for 48 hours. DNA content, cell cycle analyses, and apoptosis were done in a Guava EasyCyte™ (Merck Millipore, Darmstadt, Germany). For IF assays, MCF-7/Pit-1 cells were cultured on glass coverslips for 24 hours, treated with the drugs for 48 hours, and incubated overnight with an anti-pH2AX antibody. Evaluation of DNA fragmentation by comet assay was performed using a protocol for the single cell gel electrophoresis as previously described [42]. The average comet tail length is indicated.

### Animal studies

Twenty four female mice, age-matched between 6–8 weeks, homozygous for the severe combined immune deficiency spontaneous mutation (CB17-Prkdcscid, named SCID, PRBB, Barcelona, Spain) were used for xenografting studies. SCID mice were injected subcutaneously into the left and right flanks with 7 × 10^6^ MCF-7/Pit-1-luc (24 mice, 48 tumors). Fifteen days after cells injection, mice were randomized into 4 groups of 6 mice each. One group was treated intraperitoneally (i.p.) with 3-Epi every other day (0.5 μg/kg weight, dissolved in sesame oil). Another group was treated only twice with cisplatin (7 mg/kg weight, i.p., days 0 and 7 of treatment). A third group was treated with 3-Epi every other day (i.p., 0.5 μg/kg weight) + cisplatin only twice (7 mg/kg weight, i.p., days 0 and 7 of treatment). Finally, a control group was treated with vehicle (sesame oil). Tumor growth was monitored every 7 days from day 15 (0 day of treatment) until day 36 (21 days of treatment) using *in Vivo* Imaging System (IVIS, Caliper Life Sciences, Alameda, USA), and tumor volume was calculated. An intensity map was obtained using the Living Image software (Caliper Life Sciences). The software uses a color-based scale to represent the intensity of each pixel (ranging from blue representing low to red representing high).

Calcium levels were evaluated in male swiss CD-1 mice. Mice (5 per group) were injected intraperitoneally at several doses (0.1, 0.3, 0.5, or 1 μg/kg weight) either with 1, 25D or 3-Epi dissolved in sesame oil every other day for three weeks. Control group was injected with sesame oil. Calcium in serum was determined using the QuantiChom Calcium Assay Kit (BioAssay Systems, Hayward, CA, USA). Calcium levels were also evaluated in the mice xenograft tumor model after treatments.

### Statistical analysis

Comparison of continuous variables among groups was done using a two-sided Student *t* test or 1-way ANOVA, with the Tukey-Kramer multiple comparison test for post-hoc comparisons. At least two independent experiments were performed. Correlation between variables was evaluated using the Spearman's rank correlation test. Differences were considered to be statistically significant when *P* < 0.05. All data were analyzed using the statistical software SPSS 15.0 (SPSS, Inc, Chicago, IL).

## SUPPLEMENTARY DATA



## References

[1] Perou CM, Sørlie T, Eisen MB, van de Rijn M, Jeffrey SS, Rees CA, Pollack JR, Ross DT, Johnsen H, Akslen LA, Fluge O, Pergamenschikov A, Williams C (2000). Molecular portraits of human breast tumours. Nature.

[2] Sørlie T, Perou CM, Tibshirani R, Aas T, Geisler S, Johnsen H, Hastie T, Eisen MB, van de Rijn M, Jeffrey SS, Thorsen T, Quist H, Matese JC (2001). Gene expression patterns of breast carcinomas distinguish tumor subclasses with clinical implications. Proc Natl Acad Sci USA.

[3] Herschkowitz JI, Simin K, Weigman VJ, Mikaelian I, Usary J, Hu Z, Rasmussen KE, Jones LP, Assefnia S, Chandrasekharan S, Backlund MG, Yin Y, Khramtsov AI (2007). Identification of conserved gene expression features between murine mammary carcinoma models and human breast tumors. Genome Biol.

[4] Kennecke H, Yerushalmi R, Woods R, Cheang MC, Voduc D, Speers CH, Nielsen TO, Gelmon K (2010). Metastatic behavior of breast cancer subtypes. J Clin Oncol.

[5] Lam SW, Jimenez CR, Boven E (2014). Breast cancer classification by proteomic technologies: Current state of knowledge. Cancer Treat Rev.

[6] Santagata S, Thakkar A, Ergonul A, Wang B, Woo T, Hu R, Harrell JC, McNamara G, Schwede M, Culhane AC, Kindelberger D, Rodig S, Richardson A, Schnitt SJ (2014). Taxonomy of breast cancer based on normal cell phenotype predicts outcome. J Clin Invest.

[7] Haricharan S, Bainbridge MN, Scheet P, Brown PH (2014). Somatic mutation load of estrogen receptor-positive breast tumors predicts overall survival: an analysis of genome sequence data. Breast Cancer Res Treat.

[8] Tanioka M, Sasaki M, Shimomura A, Fujishima M, Doi M, Matsuura K, Sakuma T, Yoshimura K, Saeki T, Ohara M, Tsurutani J, Watatani M (2014). Pathologic complete response after neoadjuvant chemotherapy in HER2-overexpressing breast cancer according to hormonal receptor Status. Breast.

[9] Rakha EA, Reis-Filho JS, Ellis IO (2008). Basal-Like Breast Cancer: A Critical Review. J Clin Oncol.

[10] Futreal PA, Coin L, Marshall M, Down T, Hubbard T, Wooster R, Rahman N, Stratton MR (2004). A census of human cancer genes. Nat Rev Cancer.

[11] Koboldt DC, Fulton RS, McLellan MD, Schmidt H, Kalicki-Veizer J, McMichael JF, Fulton LL, Dooling DJ, Ding L, Mardis ER, Wilson RK, Ally A, Balasundaram M (2012). Comprehensive molecular portraits of human breast tumours. Nature.

[12] Liu C, Srihari S, Cao KA, Chenevix-Trench G, Simpson PT, Ragan MA, Khanna KK (2014). A fine-scale dissection of the DNA double-strand break repair machinery and its implications for breast cancer therapy. Nucleic Ac Res.

[13] Furuta S, Jiang X, Gu B, Cheng E, Chen P-L, Lee W-H (2005). Depletion of BRCA1 impairs differentiation but enhances proliferation of mammary epithelial cells. Proc Natl Acad Sci USA.

[14] Bai F, Smith MD, Chan HL, Pei XH (2013). Germline mutation of Brca1 alters the fate of mammary luminal cells and causes luminal-to-basal mammary tumor transformation. Oncogene.

[15] Venkitaraman AR (2014). Cancer suppression by the chromosome custodians, BRCA1 and BRCA2. Science.

[16] Feldman D, Krishnan AV, Swami S, Giovannucci E, Feldman BJ (2014). The role of vitamin D in reducing cancer risk and progression. Nat Rev Cancer.

[17] Deeb KK, Trump DL, Johnson CS (2007). Vitamin D signaling pathways in cancer: Potential for anticancer therapeutics. Nat Rev Cancer.

[18] Cho YL, Christensen C, Saunders DE, Lawrence WD, Deppe G, Malviya VK, Malone JM (1991). Combined effects of 1, 25-dihydroxyvitamin D3 and platinum drugs on the growth of MCF-7 cells. Cancer Res.

[19] Seoane S, Perez-Fernandez R (2006). The vitamin D receptor represses transcription of the pituitary transcription factor Pit-1 gene without involvement of the retinoid X receptor. Mol Endocrinol.

[20] Gil-Puig C, Seoane S, Blanco M, Macia M, Garcia-Caballero T, Segura C, Perez-Fernandez R (2005). Pit-1 is expressed in normal and tumoral human breast and regulates growth hormone secretion and cell proliferation. Eur J Endocrinol.

[21] Ben-Batalla I, Seoane S, Garcia-Caballero T, Gallego R, Macia M, Gonzalez LO, Vizoso F, Perez-Fernandez R (2010). Deregulation of the Pit-1 transcription factor in human breast cancer cells promotes tumor growth and metastasis. J Clin Invest.

[22] Reddy GS, Muralidharan KR, Okamura WH, Tserng K-Y, McLane JA, Norman AW, Bouillon R, Thomasset M (1994). Metabolism of 1a, 25-dihydroxyvitamin D3 and one of its A-ring diastereomer 1a, 25-dihydroxy-3-epivitamin D3 in neonatal human keratinocytes. Vitamin D a pluripotent steroid hormone: Structural studies, molecular endocrinology and clinical applications.

[23] Reddy GS, Rao DS, Siu-Caldera ML, Astecker N, Weiskopf A, Vouros P, Sasso GJ, Manchand PS, Uskokovic MR (2000). 1a, 25-dihydroxy-16-ene-23-yne-vitamin D3 and 1a, 25-dihydroxy-16-ene-23-yne-20-epi-vitamin D3: analogs of 1a, 25-dihydroxyvitamin D3 that resist metabolism through the C-24 oxidation pathway are metabolized through the C-3 epimerization pathway. Arch Biochem Biophys.

[24] Buras RR, Schumaker LM, Davoodi F, Brenner RV, Shabahang M, Nauta RJ, Evans SRT (1994). Vitamin D receptors in breast cancer cells. Breast Cancer Res Treat.

[25] Turashvili G, Bouchal J, Baumforth K, Wei W, Dziechciarkova M, Ehrmann J, Klein J, Fridman E, Skarda J, Srovnal J, Hajduch M, Murray P, Kolar Z (2007). Novel markers for differentiation of lobular and ductal invasive breast carcinomas by laser microdissection and microarray analysis. BMC Cancer.

[26] Richardson AL, Wang ZC, De Nicolo A, Lu X, Brown M, Miron A, Liao X, Iglehart JD, Livingston DM, Ganesan S (2006). X chromosomal abnormalities in basal-like human breast cancer. Cancer Cell.

[27] Alimonti A, Carracedo A, Clohessy JG, Trotman LC, Nardella C, Egia A, Salmena L, Sampieri K, Haveman WJ, Brogi E, Richardson AL, Zhang J, Pandolfi PP (2010). Subtle variations in Pten dose determine cancer susceptibility. Nat Genet.

[28] Holt JT, Thompson ME, Szabo C, Robinson-Benion C, Arteaga CL, King M-Cl, Jensen RA (1996). Growth retardation and tumour inhibition by BRCA1. Nature Genet.

[29] Byrski T, Huzarski T, Dent R, Marczyk E, Jasiowka M, Gronwald J, Jakubowicz J, Cybulski C, Wisniowski R, Godlewski D, Lubinski J, Narod SA (2014). Pathologic complete response to neoadjuvant cisplatin in BRCA1-positive breast cancer patients. Breast Cancer Res Treat.

[30] Molnar F, Sigueiro R, Sato Y, Araujo C, Schuster I, Antony P, Peluso J, Muller C, Mouriño A, Moras D, Rochel N (2011). 1α, (OH)2–3-Epi-vitamin D3, a natural physiological metabolite of vitamin D3: Its synthesis, biological activity and crystal structure with its receptor. PLoS One.

[31] Ma Y, Trump DL, Johnson CS (2010). Vitamin D in combination cancer treatment. J Cancer.

[32] Ma Y, Yu WD, Hershberger PA, Flynn G, Kong RX, Trump DL, Johnson CS (2008). 1, 25D3 potentiates cisplatin antitumor activity by p73 induction in a squamous cell carcinoma model. Mol Cancer Ther.

[33] Smith DC, Johnson CS, Freeman CC, Muindi J, Wilson JW, Trump DL (1999). A Phase I trial of calcitriol (1, 25-dihydroxycholecalciferol) in patients with advanced malignancy. Clin Cancer Res.

[34] Fakih MG, Trump DL, Muindi JR, Black JD, Bernardi RJ, Creaven PJ, Schwartz J, Brattain MG, Hutson A, French R, Johnson CS (2007). A phase I pharmacokinetic and pharmacodynamic study of intravenous calcitriol in combination with oral gefitinib in patients with advanced solid tumors. Clin Cancer Res.

[35] Rhieu SY, Annalora AJ, Wang G, Flarakos CC, Gathungu RM, Vouros P, Sigüeiro R, Mouriño A, Schuster I, Palmore GT, Reddy GS (2013). Metabolic stability of 3-Epi-1α, 25-Dihydroxyvitamin D3 over 1α, 25-Dihydroxyvitamin D3: metabolism and molecular docking studies using rat CYP24A1. J Cell Biochem.

[36] Byrski T, Dent R, Blecharz P, Foszczynska-Kloda M, Gronwald J, Huzarski T, Cybulski C, Marczyk E, Chrzan R, Eisen A, Lubinski J, Narod SA (2012). Results of a phase II open-label, non-randomized trial of cisplatin chemotherapy in patients with BRCA1-positive metastatic breast cancer. Breast Cancer Res.

[37] Campbell MJ, Reddy SG, Koeffler HP (1997). Vitamin D3 analogs and their 24-oxo metabolites equally inhibit clonal proliferation of a variety of cancer cells but have differing molecular effects. J Cell Biochem.

[38] Cambell MJ, Gombart AF, Kwok SH, Park S, Koeffler HP (2000). The antiproliferative effects of 1alpha, (OH)2D3 on breast and prostate cancer cells are associated with induccion of BRCA1 gene expression. Oncogene.

[39] Pickholtz I, Saadyan S, Keshet GI, Wang VS, Cohen R, Bouwman P, Jonkers J, Byers SW, Papa MZ, Yarden RI (2014). Cooperation between BRCA1 and vitamin D is critical for histone acetylation of the p21waf1 promoter and for growth inhibition of breast cancer cells and cancer stem-like cells. Oncotarget.

[40] Huang Y-L, Chou W-C, Hsiung C-N, Hu L-Y, Chu H-W, Shen C-Y (2015). FGFR2 regulates Mre11 expression and double-strand break repair via the MEK-ERK-POU1F1 pathway in breast tumorigenesis. Hum Mol Genet.

[41] Seoane S, Diaz-Rodriguez P, Sendon-Lago J, Gallego R, Perez-Fernandez R, Landin M (2013). Administration of the optimized β-Lapachone-poloxamer-cyclodextrin ternary system induces apoptosis, DNA damage and reduces tumor growth in a human breast adenocarcinoma xenograft mouse model. Eur J Pharm Biopharm.

[42] Seoane S, Montero JC, Ocaña A, Pandiella A (2010). Effect of multikinase inhibitors on caspase-independent cell death and DNA damage in HER2-overexpressing breast cancer cells. J Natl Cancer Inst.

