# Supplementary material for: Pit-1 inhibits BRCA1 and sensitizes human breast tumors to cisplatin and vitamin D treatment
Source: Oncotarget. 2015 May 8;6(16):14456–71. doi: 10.18632/oncotarget.3894 (PMC4546479; doi:10.18632/oncotarget.3894)
Supplement: Supplementary file 1 [file oncotarget-06-14456-s001.pdf]

SUPPLEMENTARY DATA

Reagents

Mother solutions of 1α, 25-dihydroxyvitamin D_3 (1, 25D) and 1α, 25-dihydroxy-3-epi-vitamin D_3 (3-Epi) were prepared with ethanol (95%) and their concentrations were established by UV. ^{1}H- and ^{13}C-NMR spectra together with high resolution mass spectrometry were done to verify their structure. Purity of these compounds was evaluated by HPLC to be greater than 99%.

Cell lines, three-dimensional (3-D) cultures, primary cultures, treatments, and breast tumors cDNA samples

Human breast adenocarcinoma cell lines MCF-7, T47D, BT474, SKBR3, Hs578T, MDA-MB-231, HBL100, HCC1937, HCC1187, MCF-7/Pit-1 (stably or transfected transiently with the pcDNA3-Pit-1 overexpression vector), and MDA-MB-231/shPit-1 (stably transfected with the pLKO-shPit-1 lentiviral vector for Pit-1 knock-down, see below) were grown as previously described (1). Breast cancer cell lines and primary cultures were treated for 48 h with ethanol (control cells), 3-Epi (10, 100 and 500 nM), cisplatin (1, 5, and 10 µM), and 3-Epi+cisplatin (100 nM+5 µM) in the MTT assay. In all other *in vitro* experiments, 3-Epi was used at 100 nM, cisplatin at 5 µM, and 3-Epi+cisplatin at doses of 100 nM+5 µM, respectively, unless specifically indicated. Cisplatin and 3-Epi were diluted in PBS and ethanol, respectively. Treatment of cells with UV radiation was carried out by exposure to 150 J/m2 of UV radiation for 30 min.

For 3D culture, slides were coated with 80 µl of ice-cold matrigel (BD Biosciences) and incubated at 37ºC to allow the matrigel to solidify. A trypsinized single cell suspension containing 1000 cells in a 100 µl volume of the medium supplemented with 5% (v/v) of matrigel was carefully loaded on top of the solidified matrigel, and the plate was incubated at 37ºC for 30 minutes to allow the cells to attach to the matrigel. Then, 400 µl of the medium were added per well, and the cells were cultured for 10 days. Cells were then treated for 7 days with 3-Epi, cisplatin and 3-Epi +cisplatin. Media containing treatments were refreshed every 2 days. The spheroids were stained with DAPI and fluorescence and phase contrast photographs were taken with one inverted microscope (Olympus IX51, Hamburg, Germany). The microscope was equipped with a camera and the CellSens 1.4 Imaging software, purchased from Olympus. Quantization of the sphere diameters was performed manually by tracing a straight line across the diameter of the sphere and scoring its value as arbitrary length units. Twenty spheres were scored for each condition.

Primary cultures were obtained from breast tumors of women treated at Fundación Hospital de Jove (Gijón, Spain) according to our institutional guidelines. The study adhered to national regulations and was approved by our institution's Ethics and Investigation Committee. Breast carcinoma tissue samples were obtained at the time of surgery. Prior informed consent was obtained from patients. After tumor resection, the pathologist examined and obtained a representative piece of tumor tissue. The tumor was mechanically disaggregated by mincing with scalpel and scissors to 1–2 mm^3 in a 6 well plate. Tissue was digested with 1.25 mg/ml of collagenase A in complete-DMEM-F12 medium for 48 hours in an air-CO_2 (95:5) atmosphere at 37ºC. For 7 days, primary cultures were grown in a DMEM-F12 medium, supplemented with 10% FBS, 100 U/ml penicillin, and 100 µg/ml streptomycin, 10 ng/ml epidermal growth factor (EGF), 0.5 µg/ml hydrocortisone, and 10 µg/ml insulin. Afterwards, cells were cultured at 1500 2000 cells/ cm^2 in a DMEM-F12 medium supplemented with 10% FBS, 100 U/ml penicillin, and 100 µg/ml streptomycin. Treatments and doses were as described above, patient and tumor characteristics are shown in Supplementary Table S1.

Real-time PCR, PCR, mRNA microarray, and Western blot

The primer sequences used in real-time PCR were as follows:

	Oligonucleotide sequences
Pit-1	Fw: 5′-GTGTCTACCAGTCTCCAACC-3′<br>Rv: 5′-ACTTTTCCGCCTGAGTTCCT-3
BRCA1	Fw: 5′- TGTGCTTTTCAGCTTGACACAGG-3′<br>Rev: 5′- CGTCTTTTGAGGTTGTATCCGCTG-3′
VDR	Fw: 5′-GACTTTGACCGGAACGTGCC-3′<br>Rv: 5′- CATCATGCCGATGTCCACAC-3′
18S	Fw: 5′-GTAACCCGTTGAACCCCATT-3′<br>Rv: 5′-CCATCCAATCGCTA GTAGCG-3′
RAD1	Fw: 5′-AATGCAAAGTGTGTGCAAGC-3′<br>Rv: 5′-GTCCCTGGCATAGGACTTGA-3′
RAD18	Fw: 5′-TTGAATTTTGCACGGAATCA-3′<br>Rv: 5′-ATTAACCTGCTCCCCTGCTT-3′
RAD51	Fw: 5′-TTTGGAGAATTCCGAACTGG-3′<br>Rv: 5′-CATCACTGCCAGAGAGACCA-3′
RAD52	Fw: 5′-AGTTTTGGGAATGCACTTGG-3′<br>Rv: 5′-TCGGCAGCTGTTGTATCTTG-3′
RAD54B	Fw: 5′-GAGGGCCAAACACTGATGAT-3′<br>Rv: 5′-AAACATTTCCTGGCAACCTG-3′
GADD45A	Fw: 5′-GGAGGAAGTGCTCAGCAAAG-3′<br>Rv: 5′-ATCTCTGTCGTCGTCCTCGT-3′
GADD45B	Fw: 5′-ACAGTGGGGGTGTACGAGTC-3′<br>Rv: 5′-GGATGAGCGTGAAGTGGATT-3′
GADD45G	Fw: 5′-TACGCTGATCCAGGCTTTCT-3′<br>Rv: 5′-TTCGAAATGAGGATGCAGTG-3′

Full length Pit-1 cDNA was synthesized by PCR. Oligonucleotides containing Bam HI and Xho I restriction sequences were:

	Oligonucleotide sequences
Pit-1	Fw: 5′-CGCGGATCCATGAGTTGCCAAGCTTTTACT-3′<br>Rv: 5′-CGCGGATCCAATAAACCTTCTTCTCAAGAG-3

The PCR product was then subcloned into Bam HI and Xho I restriction sites of the pcDNA3 vector to obtain the pcDNA3-Pit-1 overexpression vector.

For Western blots, the anti-mouse Dylight™ 680-conjugated and anti-rabbit Dylight™ 800-conjugated were used in quantitative Western blot (Thermo Fisher Scientific, Waltham, USA). Western blot signal was detected with the Pierce ECL™ Western Blotting Substrate and visualized by placing the blot in contact with standard X-ray film. The following antibodies were used:

Antigen	Source	Application
Bad	CellSignaling	WB
Bak	CellSignaling	WB
Bax	CellSignaling	WB
Bcl-2 (N-19)	Santa Cruz Biotech.	WB
Bcl-x (H-5)	Santa Cruz Biotech	WB
Bid	CellSignaling	WB
Bik	CellSignaling	WB
BRCA1 (D54A8)	CellSignaling	WB
Caspase 12	CellSignaling	WB
Caspase 12 active	CellSignaling	WB
Caspase 3 active (5A1E)	CellSignaling	WB
Caspase 8 (D35G2)	CellSignaling	WB
Caspase 8 active (18C8)	CellSignaling	WB
CDK1/2 (AN21.2)	Santa Cruz Biotech.	WB
Cleaved PARP (46D11)	CellSignaling	WB
Cyclin A	BDBiosciences	WB
Cyclin B	BDBiosciences	WB
Cyclin D1 (clone 7213G)	Santa Cruz Biotech.	WB
Cyclin D1 (H-295)	Santa Cruz Biotech.	WB
Cyclin E (M-20)	Santa Cruz Biotech.	WB
GADD45□(H-165)	Santa Cruz Biotech.	WB
GAPDH (clone 6C5)	Santa Cruz Biotech.	WB
Mcl-1 (S-19)	Santa Cruz Biotech	WB
PARP	CellSignaling	WB
p-ATM Ser1981 (D6H9)	CellSignaling	WB
p-ATR Ser428	CellSignaling	WB
p-BRCA1 Ser988	CellSignaling	WB
p-Chk1 Ser296	CellSignaling	WB
p-Chk2 Thr68	CellSignaling	WB
p-H2AX Ser139 (20E3)	CellSignaling	WB, IF
Pit-1 (X-7)	Santa Cruz Biotech.	WB, IHC, ICC
p-p53 Ser15 (16G8)	CellSignaling	WB
p-Rb Ser807/811	CellSignaling	WB
Puma	CellSignaling	WB
Rad 1 (N-18)	Santa Cruz Biotech	WB

(*Continued*)

Antigen	Source	Application
Rad 18 (H-77)	Santa Cruz Biotech.	WB
Rad 51 (H-92)	Santa Cruz Biotech.	WB
Rad 54B (N-16)	Santa Cruz Biotech.	WB
VDR (clone 9A7)	Millipore	WB, IHC

Plasmids, transfections, and luciferase reporter and chromatin immunoprecipitation (ChIP) assays

Stable transfection of the MCF-7 Tet-Off cells (Clontech) with the pTRE2 control vector and the pTRE2-hPit-1 overexpression vector was performed as previously described [1] by electroporation with the Nucleofector apparatus (Amaxa biosystems) using the VCA-1003 (Cell line Nucleofector Kit V, Amaxa biosystems). Stable transfectants of MCF-7 Tet-Off cells were isolated as single colonies following selection in G418 (500 µg/ml). Stable clones of Pit-1 knock-down in MDA-MB-231 cells (MDA-MB-231/shPit-1) were performed by transfecting cells with a mix of pLKO-shPit-1 lentiviral vectors containing three different short hairpin RNA (shRNA) sequences for Pit-1 (obtained from Santa Cruz Biotech). A mix of three different pLKO-scrambled were used as negative control in knock-down experiments (Santa Cruz Biotech). To select stable clones, 48 hours after transfection, cells were treated with 2.5 µg/µl of puromicin. Stable Pit-1 overexpressing MCF-7 (MCF-7/Pit-1) cells, and MDA-MB-231 cells were also transfected with pBABE-puro-Luc vector and 48 hours later treated with 2.5 µg/µl of puromicin to select clones (MCF-7/Pit-1-luc and MDA-MB-231-luc cells).

For luciferase reporter assay, MCF-7 cells were cultured for 24 h in DMEM supplemented with 10% of charcoal-stripped FCS, culture medium was replaced with phenol red free DMEM containing 10% FCS for 24 h with 3-Epi and 1, 25D at several concentrations (1×10^{-11} to 1×10^{-6} M). Cells were then treated for 10 min with luciferin potassium salt (100 mg/L) (Regis Technologies, Morton Grove, USA), and bioluminescence images were taken with the *In Vivo* Imaging System (IVIS, Caliper Life Sciences, Alameda, CA, USA), quantified as total photon counts, and processed by Living Image software (Caliper Life Sciences). The EC_{50} values are derived from dose-response curves and represent the analogue concentration capable of increasing luciferase activity by 50%. The luciferase activity ratio is the average ratio of the EC_{50} for the analogue to the EC_{50} for 1, 25D.

For BRCA1 reporter assays, MCF-7 cells were transfected in 6-well plates containing 6 µl of jet PEI Polyplus transfection reagent, 1 µg of each reporter plasmid, and 50 ng of pRL-TK-Renilla (as transfection control) for 48 hours. The cells were lysed in 5 µl lysis buffer (Promega, Madison, USA) and luciferase activity was then measured in a Mithras LB 940 apparatus (Berthold Technologies) using Dual-Luciferase Reporter Assay System kit (Promega). The proximal promoter regions of the human BRCA1 gene (pGL3B-hBRCA1$_{-1520/+1}$, pGL3B-hBRCA1$_{-1086/+1}$, pGL3B-hBRCA1$_{-652/+1}$) were synthesized by PCR and the product subcloned in Xho I and Hind III sites of the pGL3Basic vector. The mutant pGL3B- hBRCA1$_{1520/+1mut}$vector was constructed using site-directed mutagenesis (QuikChange kit, Stratagene, La Jolla, USA) under the conditions recommended by the manufacturer. Mutations are located in positions −1031, −1028, −1027 bp from the start transcription site. All plasmids were checked by sequencing. The primer sequences used are listed below. The mutagenized oligonucleotide is identified by lowercase letters.

	Oligonucleotide sequences
pGL3B-hBRCA1$_{-1520/+1}$	Fw: 5′-CCCCTCGAGCCCGCGCCCACCTGGAAC-3
pGL3B-hBRCA1$_{-1086/+1}$	Fw: 5′-CCCCTCGAGCTAGCTAACCCAGGCTGCTTC-3′
pGL3B-hBRCA1$_{-652/+1}$	Fw: 5′-CCCCTCGAGTTGTACGTATCTTTTTAA-3′
pGL3B-hBRCA1$_{-1520/+1\ mut}$	Fw: 5′-TGGGGAGGCGGCAcTGCccAGACCGTCCGCT- 3′
Reverse primer (−20/−1)	Rv: 5′-CCCTTCGAACGAAGAGCAGATAAATCCAT-3′

In the ChIP assay, PCR was run for 60 sec at 95°C, 58°C, and 72°C within each cycle, for 35 cycles in total. The three pairs of BRCA1 primers were as follows: (A) (−1082/−1062 bp from transcription start site), and reverse (−895/−1015 bp), PCR product was 187 bp long; (B) forward (−471/−451 bp), and reverse (−286/−306bp), PCR product was 185 bp long; (C) forward (−74/−54 bp), and reverse (+117/ +97bp), PCR product was 196 bp long. Primer sequences were as follows:

	Oligonucleotide sequences
BRCA-1	**(A)** Fw: 5′-TGACGTGACCCCACCCCTAG-3′<br>Rv: 5′-AGAGCCAATCTTGTTGGCGA-3′
	**(B)** Fw: 5′-AACACTGTGGCGAAGACCT-3′<br>Rv: 5′-GAAGGTGAGAATCGCTACC-3′
	**(C)** Fw: 5′-CTGAGAGGCTGCTGCTTAGC-3′<br>Rv: 5′-TTTACCCAGAGCAGAGGGTG-3′

REFERENCES

1. Ben-Batalla I, Seoane S, Garcia-Caballero T, Gallego R, Macia M, Gonzalez LO, Vizoso F, Perez-Fernandez R. Deregulation of the Pit-1 transcription factor in human breast cancer cells promotes tumor growth and metastasis. J Clin Invest. 2010; 120:4289–4302.

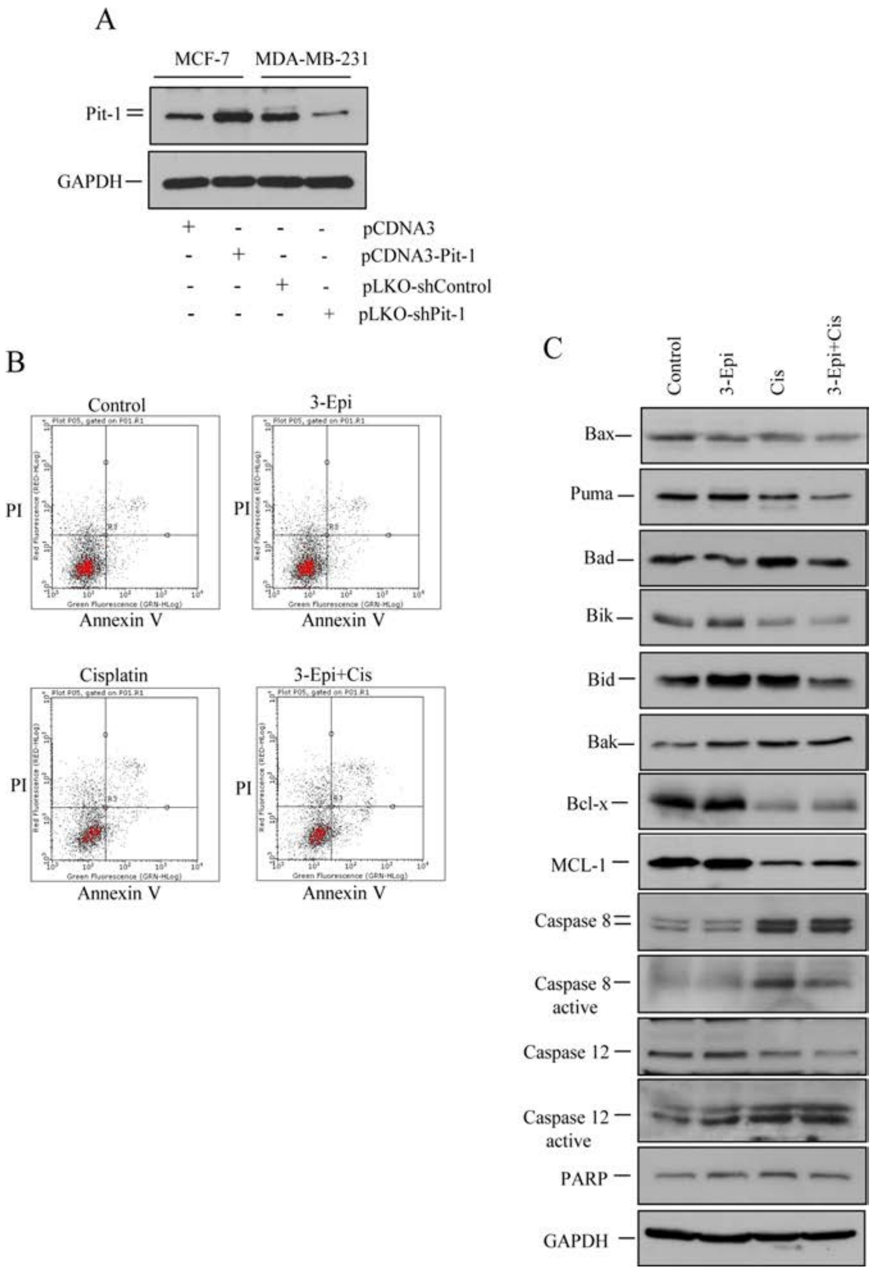

Supplementary Figure S1: A. Western blot of Pit-1 expression in MCF-7 cells (control and overexpressing Pit-1), and MDA-MB-231 cells (control and Pit-1 knockdown). GAPDH was used as loading control. **B.** 3-Epi + cisplatin increase apoptosis in stable Pit-1 overexpressing MCF-7 cells (MCF-7/Pit-1). (A) MCF-7/Pit-1 cells were treated with 100 nM of 3-Epi, 5 µM of cisplatin, or both drugs at the same doses for 48 hours, and apoptosis was examined by flow cytometry after staining with Annexin V/Propidium iodide (PI). A representative experiment is shown. C. MCF-7/Pit-1 cells were treated with the indicated drugs (as above) for 48 hours, and an immunoblot analysis was done for the anti-apoptotic (Bcl-x, MCL-1) and pro-apoptotic (Bax, Puma, Bad, $pBad_{Ser112}$, Bik, Bid, Bak, Caspase 8, Caspase 8 active, Caspase 12, Caspase 12 active, PARP) proteins. GAPDH was used as loading control.

A

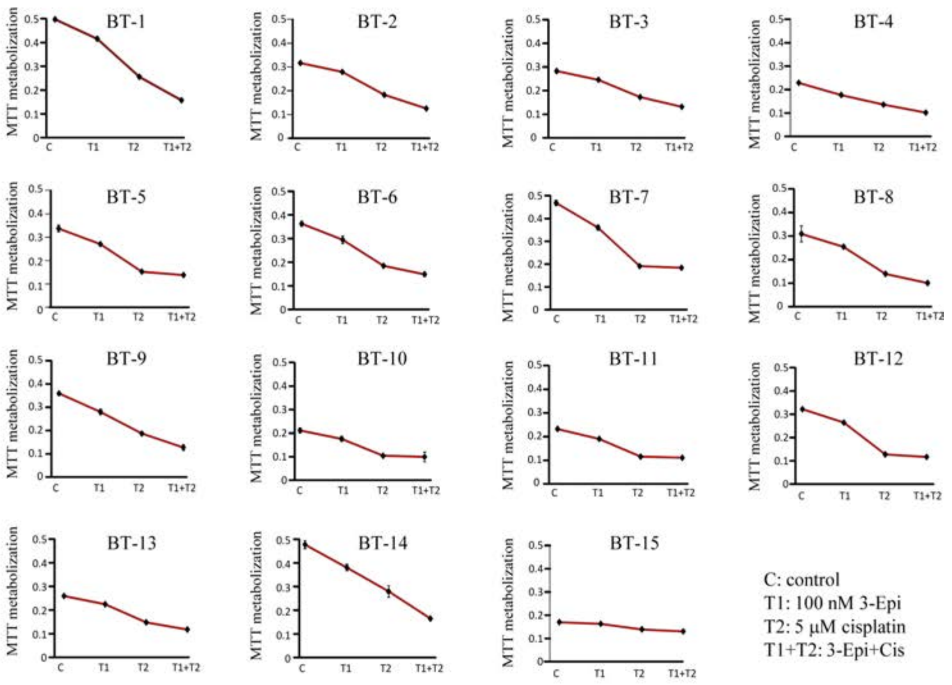

B

	C	T1	T2	T1+T2
**BT-1**	0.496 ± 0.007	0.415 ± 0.007	0.255 ± 0.007	0.157 ± 0.007
**BT-2**	0.316 ± 0.006	0.278 ± 0.006	0.182 ± 0.007	0.125 ± 0.005
**BT-3**	0.282 ± 0.004	0.245 ± 0.002	0.172 ± 0.006	0.131 ± 0.005
**BT-4**	0.228 ± 0.004	0.176 ± 0.006	0.135 ± 0.004	0.101 ± 0.006
**BT-5**	0.228 ± 0.014	0.270 ± 0.004	0.152 ± 0.005	0.138 ± 0.005
**BT-6**	0.336 ± 0.01	0.294 ± 0.01	0.184 ± 0.007	0.149 ± 0.009
**BT-7**	0.362 ± 0.011	0.359 ± 0.011	0.191 ± 0.004	0.184 ± 0.006
**BT-8**	0.289 ± 0.033	0.254 ± 0.008	0.139 ± 0.001	0.100 ± 0.007
**BT-9**	0.359 ± 0.006	0.28 ± 0.012	0.187 ± 0.004	0.127 ± 0.012
**BT-10**	0.211 ± 0.010	0.175 ± 0.009	0.104 ± 0.006	0.099 ± 0.02
**BT-11**	0.232 ± 0.006	0.190 ± 0.004	0.115 ± 0.004	0.110 ± 0.005
**BT-12**	0.321 ± 0.006	0.264 ± 0.007	0.127 ± 0.002	0.116 ± 0.004
**BT-13**	0.259 ± 0.001	0.224 ± 0.003	0.147 ± 0.001	0.117 ± 0.001
**BT-14**	0.256 ± 0.017	0.38 ± 0.013	0.279 ± 0.024	0.164 ± 0.007
**BT-15**	0.17 ± 0.001	0.162 ± 0.004	0.138 ± 0.007	0.130 ± 0.005

Supplementary Figure S2: A. Fifteen primary cultures of human breast tumors (BT) were treated for 48 h with ethanol (controls, C), 100 nM of 3-Epi (T1), 5 μM of cisplatin (T2), or 100 nM of 3-Epi + 5 μM of cisplatin (T1 + T2). Then, MTT was added and absorbance measured at 570 nm. B. Absorbance values are represented as the mean ± SD of quadruplicate values.

Supplementary Table S1. Characteristics of human breast tumors (BT) used in primary cultures

Tumors	Age	Hist gr SBR	Ki67	TNM	ER	PR	HER2/neu
BT-1	56	1	7%	T1cN0	(+++)	(+++)	NEGATIVE
BT-2	58	2	9%	T1cN1a	(+++)	(+++)	NEGATIVE
BT-3	63	1	7%	T1bN0	(+++)	(+++)	NEGATIVE (1+)
BT-4	50	3	60%	T2N0	(++)	(+++)	POSITIVE (3+)
BT-5	73	2	>30%	T2N0	(+)	(+)	NEGATIVE
BT-6	61	2	30%	T3N3a	(+)	(+)	POSITIVE (3+)
BT-7	33	2	>25%	T2N1	(+)	(+)	NEGATIVE (1+)
BT-8	59	1	<20%	T1bN0	(+)	(+)	NEGATIVE
BT-9	82	1	10%	T2N3	(+++)	(+++)	NEGATIVE
BT-10	60	3	65%	T2N2	(+++)	(+++)	BORDERLINE (2+)
BT-11	51	2	22%	T2N0	(+)	(+)	BORDERLINE (2+)
BT-12	79	3	18%	T2N0	(+++)	(−)	POSITIVE (3+)
BT-13	69	3	30%	T2N2	(+++)	(−)	BORDERLINE (−)
BT-14	79	3	80%	T3N1	(−)	(−)	NEGATIVE
BT-15	81	3	45%	T1cN3	(+++)	(+++)	NEGATIVE
